# Supplementary material for: Modeling glioblastoma heterogeneity as a dynamic network of cell states
Source: Mol Syst Biol. 2021 Sep 16;17(9):e10105. doi: 10.15252/msb.202010105 (PMC8444284; doi:10.15252/msb.202010105)
Supplement: Supplementary file 5 — Source Data for Figure 3 [file MSB-17-e10105-s001.zip › Figure3A_sourcedata/GSEA_3065/nosigdb_state2.GseaPreranked.1622718419215/NB_KI_CLUSTER5.html]

Details for gene set NB\_KI\_CLUSTER5[GSEA]

|  || Dataset | state2 |
| Phenotype | NoPhenotypeAvailable |
| Upregulated in class | na\_pos |
| GeneSet | NB\_KI\_CLUSTER5 |
| Enrichment Score (ES) | 0.93130964 |
| Normalized Enrichment Score (NES) | 3.0121236 |
| Nominal p-value | 0.0 |
| FDR q-value | 0.0 |
| FWER p-Value | 0.0 |
Table: GSEA Results Summary

  

Fig 1: Enrichment plot: NB\_KI\_CLUSTER5      
 Profile of the Running ES Score & Positions of GeneSet Members on the Rank Ordered List

  

| PROBE | GENE SYMBOL | GENE\_TITLE | RANK IN GENE LIST | RANK METRIC SCORE | RUNNING ES | CORE ENRICHMENT || 1 | HIST1H4C |  |  | 0 | 1.309 | 0.0396 | Yes |
| 2 | PCLAF |  |  | 2 | 0.852 | 0.0653 | Yes |
| 3 | HMGB2 |  |  | 4 | 0.823 | 0.0901 | Yes |
| 4 | H2AFZ |  |  | 8 | 0.735 | 0.1120 | Yes |
| 5 | DHFR |  |  | 11 | 0.644 | 0.1313 | Yes |
| 6 | UBE2T |  |  | 12 | 0.643 | 0.1508 | Yes |
| 7 | MCM7 |  |  | 13 | 0.626 | 0.1698 | Yes |
| 8 | PCNA |  |  | 16 | 0.595 | 0.1876 | Yes |
| 9 | CDK1 |  |  | 18 | 0.577 | 0.2049 | Yes |
| 10 | UBE2S |  |  | 19 | 0.574 | 0.2223 | Yes |
| 11 | ATAD2 |  |  | 21 | 0.563 | 0.2392 | Yes |
| 12 | CKS1B |  |  | 23 | 0.554 | 0.2559 | Yes |
| 13 | DUT |  |  | 24 | 0.552 | 0.2726 | Yes |
| 14 | HMGB1 |  |  | 25 | 0.549 | 0.2892 | Yes |
| 15 | NUDT1 |  |  | 26 | 0.541 | 0.3056 | Yes |
| 16 | CLSPN |  |  | 27 | 0.536 | 0.3218 | Yes |
| 17 | CKS2 |  |  | 28 | 0.532 | 0.3380 | Yes |
| 18 | DEK |  |  | 31 | 0.526 | 0.3537 | Yes |
| 19 | HMGN2 |  |  | 35 | 0.512 | 0.3689 | Yes |
| 20 | GINS2 |  |  | 37 | 0.506 | 0.3841 | Yes |
| 21 | HIST1H1C |  |  | 38 | 0.498 | 0.3992 | Yes |
| 22 | SMC2 |  |  | 39 | 0.493 | 0.4141 | Yes |
| 23 | DBF4 |  |  | 41 | 0.492 | 0.4289 | Yes |
| 24 | SMC4 |  |  | 42 | 0.489 | 0.4437 | Yes |
| 25 | CENPK |  |  | 44 | 0.488 | 0.4584 | Yes |
| 26 | ZWINT |  |  | 45 | 0.488 | 0.4732 | Yes |
| 27 | ANP32B |  |  | 48 | 0.474 | 0.4873 | Yes |
| 28 | KPNA2 |  |  | 50 | 0.472 | 0.5015 | Yes |
| 29 | MCM3 |  |  | 51 | 0.464 | 0.5155 | Yes |
| 30 | TUBA1B |  |  | 52 | 0.462 | 0.5295 | Yes |
| 31 | CENPM |  |  | 54 | 0.460 | 0.5433 | Yes |
| 32 | CHAF1A |  |  | 55 | 0.457 | 0.5572 | Yes |
| 33 | BIRC5 |  |  | 61 | 0.443 | 0.5700 | Yes |
| 34 | PTTG1 |  |  | 63 | 0.439 | 0.5832 | Yes |
| 35 | GMNN |  |  | 64 | 0.435 | 0.5964 | Yes |
| 36 | RANBP1 |  |  | 65 | 0.430 | 0.6094 | Yes |
| 37 | HIST1H1E |  |  | 66 | 0.429 | 0.6224 | Yes |
| 38 | FBXO5 |  |  | 67 | 0.420 | 0.6351 | Yes |
| 39 | EZH2 |  |  | 70 | 0.417 | 0.6475 | Yes |
| 40 | SGO2 |  |  | 77 | 0.406 | 0.6592 | Yes |
| 41 | NUCKS1 |  |  | 78 | 0.404 | 0.6714 | Yes |
| 42 | H2AFV |  |  | 82 | 0.397 | 0.6831 | Yes |
| 43 | MCM5 |  |  | 83 | 0.394 | 0.6951 | Yes |
| 44 | BRCA1 |  |  | 92 | 0.381 | 0.7057 | Yes |
| 45 | RAN |  |  | 98 | 0.375 | 0.7166 | Yes |
| 46 | CDT1 |  |  | 108 | 0.368 | 0.7267 | Yes |
| 47 | RHEB |  |  | 110 | 0.362 | 0.7376 | Yes |
| 48 | DNMT1 |  |  | 121 | 0.345 | 0.7470 | Yes |
| 49 | TUBB4B |  |  | 125 | 0.340 | 0.7570 | Yes |
| 50 | ANP32E |  |  | 136 | 0.330 | 0.7659 | Yes |
| 51 | CKAP2 |  |  | 151 | 0.308 | 0.7737 | Yes |
| 52 | NASP |  |  | 156 | 0.306 | 0.7826 | Yes |
| 53 | HELLS |  |  | 161 | 0.304 | 0.7913 | Yes |
| 54 | ECT2 |  |  | 171 | 0.297 | 0.7994 | Yes |
| 55 | MZT1 |  |  | 174 | 0.295 | 0.8081 | Yes |
| 56 | TEX30 |  |  | 206 | 0.279 | 0.8132 | Yes |
| 57 | KIF20B |  |  | 208 | 0.278 | 0.8216 | Yes |
| 58 | CHEK1 |  |  | 224 | 0.266 | 0.8280 | Yes |
| 59 | SHCBP1 |  |  | 231 | 0.262 | 0.8353 | Yes |
| 60 | MIS18BP1 |  |  | 257 | 0.246 | 0.8401 | Yes |
| 61 | LIG1 |  |  | 259 | 0.246 | 0.8474 | Yes |
| 62 | HIST2H2AC |  |  | 267 | 0.244 | 0.8541 | Yes |
| 63 | PRR11 |  |  | 285 | 0.238 | 0.8594 | Yes |
| 64 | TRIP13 |  |  | 286 | 0.237 | 0.8666 | Yes |
| 65 | CIP2A |  |  | 304 | 0.230 | 0.8718 | Yes |
| 66 | BUB3 |  |  | 311 | 0.228 | 0.8780 | Yes |
| 67 | TCF19 |  |  | 318 | 0.226 | 0.8843 | Yes |
| 68 | DIAPH3 |  |  | 321 | 0.226 | 0.8909 | Yes |
| 69 | MXD3 |  |  | 328 | 0.224 | 0.8970 | Yes |
| 70 | RACGAP1 |  |  | 333 | 0.222 | 0.9033 | Yes |
| 71 | PLK1 |  |  | 364 | 0.213 | 0.9066 | Yes |
| 72 | CENPW |  |  | 373 | 0.211 | 0.9121 | Yes |
| 73 | ZWILCH |  |  | 374 | 0.210 | 0.9184 | Yes |
| 74 | NCAPD2 |  |  | 434 | 0.193 | 0.9180 | Yes |
| 75 | SPDL1 |  |  | 459 | 0.189 | 0.9211 | Yes |
| 76 | CDKN2D |  |  | 499 | 0.182 | 0.9225 | Yes |
| 77 | PRC1 |  |  | 514 | 0.178 | 0.9264 | Yes |
| 78 | CDC25B |  |  | 519 | 0.178 | 0.9313 | Yes |
| 79 | FANCA |  |  | 743 | 0.145 | 0.9119 | No |
| 80 | GAPDH |  |  | 933 | 0.125 | 0.8954 | No |
| 81 | CEP152 |  |  | 1047 | 0.116 | 0.8869 | No |
| 82 | DDX11 |  |  | 1310 | 0.097 | 0.8618 | No |
| 83 | CDCA7 |  |  | 1684 | 0.078 | 0.8243 | No |
| 84 | GGH |  |  | 2149 | 0.059 | 0.7765 | No |
| 85 | ARL6IP1 |  |  | 2421 | 0.050 | 0.7490 | No |
| 86 | LBR |  |  | 7064 | -0.038 | 0.2540 | No |
Table: GSEA details [plain text format]

  

Fig 2: NB\_KI\_CLUSTER5: Random ES distribution      
 Gene set null distribution of ES for **NB\_KI\_CLUSTER5**
